# 2110 Prenatal near roadway air pollution exposure and early neurodevelopment in young Mexican-American children: Findings from the CHAMACOS prospective birth cohort study

**DOI:** 10.1017/cts.2018.300

**Published:** 2018-11-21

**Authors:** William H. Coe, Jason Feinberg, Robert Grunier, Brenda Eskenazi, Heather Volk

**Affiliations:** 1 Johns Hopkins University School of Medicine; 2 Johns Hopkins Bloomberg School of Public Health; 3 UC Berkeley School of Public Health

## Abstract

OBJECTIVES/SPECIFIC AIMS: Previous studies suggest that prenatal exposure to environmental pollutants can have an adverse effect on brain development. We examine the association between prenatal near roadway air pollution (NRAP) exposure and early neurodevelopment. METHODS/STUDY POPULATION: The Center for the Health Assessment of Mothers and Children of Salinas (CHAMACOS) Study is a prospective birth cohort that began in 1999 with 605 mother-child pairs of primarily Mexican-American descent. Maternal residence during pregnancy was geocoded using ArcGIS and prenatal NRAP exposure was assigned using the CALINE4 line source dispersion model. We used composite Bayley Scale scores for cognitive and motor development, and created separate linear regression models at 6, 12, and 24 months of age. RESULTS/ANTICIPATED RESULTS: After adjusting for relevant maternal and child characteristics, preliminary estimates suggest that prenatal NRAP exposure is associated with a nonsignificant increase in Bayley Scale scores at 6 and 24 months (cognitive: β=0.13, *p*-value=0.20 and motor: β=0.08, *p*-value=0.58 at 6 months; cognitive: β=0.16, *p*-value=0.42 and motor: β=0.20, *p*-value=0.25 at 24 months) and a nonsignificant decrease at 12 months (cognitive: β=−0.07, *p*-value=0.64 and motor: β=−0.12, *p*-value=0.56). DISCUSSION/SIGNIFICANCE OF IMPACT: Our preliminary findings do not suggest that prenatal NRAP exposure is associated with early cognitive development. Additional exploration of co-exposures known to effect neurodevelopment should be examined in this rural population.

